# Specific expertise from diffuse feedback: A feedforward-gated mechanism

**DOI:** 10.1126/sciadv.aeh5972

**Published:** 2026-10-07

**Authors:** Ruining Guo, An Yan, Minggui Chen, Wu Li, Yin Yan

**Affiliations:** ^1^ State Key Laboratory of Cognitive Neuroscience and Learning & IDG/McGovern Institute for Brain Research, Beijing Normal University, Beijing 100875, China.; ^2^ College of Life Sciences, Beijing Normal University, Beijing 100875, China.; ^3^ Chinese Institute for Brain Research, Beijing, China.

## Abstract

Perceptual learning improves sensory sensitivity through practice but is typically specific to trained stimuli. Two contrasting accounts have been proposed: fine-tuning of feature-selective neurons in early sensory cortex, which raises a stability-plasticity dilemma, and reweighting of stable sensory inputs by higher-level readout. To reconcile these views, we recorded neuronal populations in macaque visual area V4 during training on a camouflaged collinear contour detection task. Behavioral improvements were strictly orientation specific, yet learning-related neural enhancements were unexpectedly widespread, regardless of neuronal tuning. Critically, these enhancements were confined to late, feedback-influenced response components, whereas early feedforward activity remained stable. These results support a feedforward-gated feedback mechanism: Learning potentiates higher-order representations that deliver diffuse feedback, broadly recruiting early cortical neurons, while behavioral specificity arises because this modulation is gated by feature-selective feedforward inputs associated with the trained stimulus. Rather than being sequestered in a single locus or process, perceptual expertise emerges from hierarchical, bidirectional interactions that enable learning while preserving core sensory stability.

## INTRODUCTION

Visual perceptual learning, the enduring improvement in sensory discrimination through practice, is a fundamental manifestation of adult cortical plasticity. Historically, a hallmark of this process is its exquisite specificity: Performance gains often fail to transfer when stimuli are rotated to different orientations (*1*–*4*) or shifted to new visual-field locations (*2*–*5*). This has long been interpreted as evidence that plasticity resides in early visual cortex, where neurons have narrow tuning and rigid topographic organization. However, subsequent research has challenged this early-locus view, demonstrating that learning can generalize across features depending on task demands (*6*–*8*) or training protocols (*9*, *10*). This suggests that higher-level decision areas or broadly tuned regions may also mediate these gains (*11*, *12*).

These conflicting findings have given rise to two prevailing, yet unresolved, opposing views of how specific learning is achieved. The first posits the fine-tuning of feature-selective neurons within early visual cortex—an account that faces an inherent stability-plasticity dilemma, as fundamental sensory filters must remain reliable for general vision. The second suggests that learning reflects the reweighting of stable sensory inputs by higher-level readout. While the latter can explain both learning specificity and transfer, it appears inconsistent with the widespread, learning-related changes documented across the visual hierarchy, including V1 (*13*–*21*), V2 (*15*, *22*, *23*), V4 (*24*–*28*), and the inferior temporal cortex (*29*). A prominent hypothesis to bridge this gap is that early cortical plasticity is not local or intrinsic, but is instead orchestrated by feedback from higher cortical regions (*19*, *20*, *28*).

Yet, this creates a puzzle. Anatomical studies show that corticocortical feedback projections are inherently divergent and diffuse (*30*–*32*), terminating on neurons with a wide array of feature preferences (*33*, *34*). If learning is driven by such broad modulation, how does the visual system maintain the feature specificity that characterizes behavioral performance?

To investigate how hierarchical interactions resolve this tension, we recorded from macaque visual area V4, a mid-tier hub essential for feature integration and shape perception. V4 is uniquely positioned to reveal this interaction, as it receives both tuned feedforward inputs from V1/V2 and extensive feedback from the temporal and frontal lobes. By analyzing the temporal dynamics and population-wide distribution of neural enhancements, we show that behavioral specificity does not require specific feedforward plasticity. Instead, we propose a “feedforward-gated feedback” framework: Specific expertise is an emergent property in which diffuse, potentiated feedback is gated by stable, feature-selective feedforward inputs.

## RESULTS

To investigate the neural correlates of perceptual learning, we trained two naïve rhesus monkeys (*Macaca mulatta*; M1 and M2) on a camouflaged contour detection task using a two-alternative forced-choice design (Fig. 1A). In each trial, monkeys were required to maintain central fixation (300 ms) before the simultaneous presentation of two stimuli for 500 ms: a contour pattern (CP) consisting of collinear bars embedded within a background of random noise, and a noise-only pattern (NP). Following a 300-ms delay, the monkeys reported the contour location via a saccade to one of two choice dots.

**Fig. 1.**
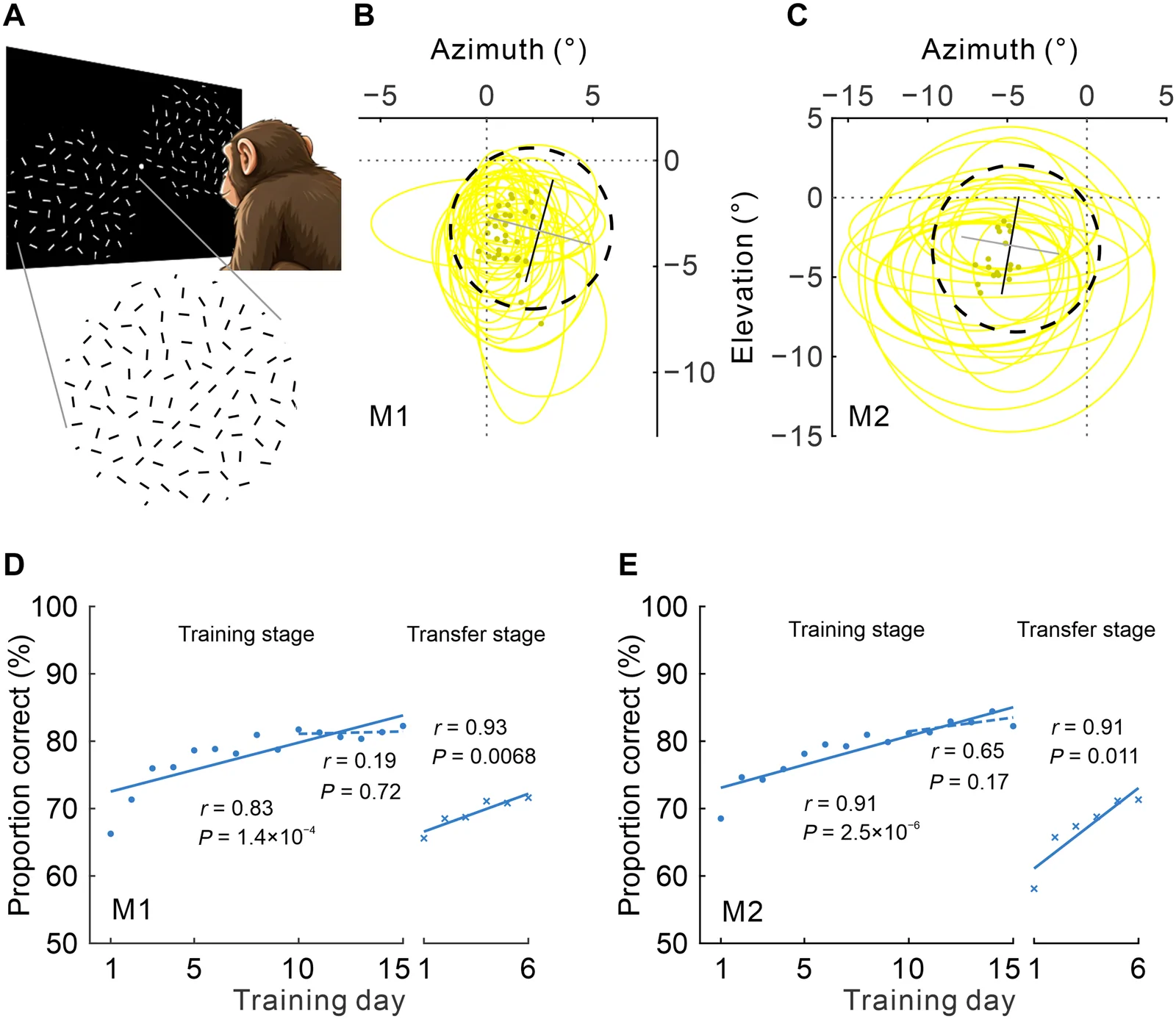
Experimental design and behavioral learning. (**A**) Illustration of the contour detection task. (**B**) Receptive fields (RFs) of recorded V4 sites in monkey M1. Yellow circles indicate RF extents (Materials and Methods), and small dots indicate RF centers. The dashed black circle marks the location of the stimulus—either the contour or noise pattern—placed over the RFs. The solid black line illustrates the contour position for the training stage; gray line, 90° rotation of the contour for the transfer stage. (**C**) Similar to (B), for monkey M2. (**D**) Behavioral accuracy of monkey M1 across training (left) and transfer (right) stages. Each data point represents the average across 36 conditions with varying task difficulty. Linear fits are shown as solid lines. The final 6 days in the training stage were analyzed separately to test for plateau (dashed line in the left panel). Pearson correlation coefficients (*r*) and *P* values (*P*) are shown. (**E**) Similar to (D), for monkey M2.

To capture the neural dynamics during learning, we chronically implanted a microelectrode array (6 × 8 electrodes, 0.4 mm spacing) in area V4 and tracked multiunit activity (MUA) at each recording site.

A block of training trials consisted of 36 unique contour configurations (Materials and Methods), covering a range of task difficulties by varying the contour’s length and the interelement spacing. On 50% of trials, the CP overlapped the cluster of recorded V4 receptive fields (RFs; Fig. 1, B and C) while the NP appeared in the opposite hemifield (Fig. 1A); this spatial arrangement was pseudorandomly interleaved across trials. This design allowed us to compare V4 population responses to an identical set of CP and NP across the trajectory of learning.

### Orientation-specific improvement in behavioral performance

To characterize the progression and specificity of learning, both monkeys were trained on a fixed collinear-contour orientation (75° for M1 and 80° for M2; Fig. 1, B and C) over a 15-day training stage. Subsequently, the contour orientation was rotated by 90° for a 6-day transfer stage to assess whether the performance gains generalized to orthogonal orientations.

During the initial training stage, contour detection accuracy improved steadily for both animals (Fig. 1, D and E, left; M1: *r =* 0.83, *P =* 1.4 × 10^−4^; M2: *r =* 0.91, *P =* 2.5 × 10^−6^; Pearson correlation, *n =* 15 days). By the final 6 days of training, performance had reached a plateau, with no further significant increases in accuracy (M1, *r =* 0.19, *P =* 0.72; M2, *r =* 0.65, *P =* 0.17; Fig. 1, D and E, dashed lines).

The onset of the transfer stage revealed a sharp behavioral cost to orientation rotation (Fig. 1, D and E, right). Accuracy dropped abruptly on the first day for the new orientation, returning to levels near the start of the primary training stage. Significant relearning was observed at the new orientation (M1: *r =* 0.93, *P =* 0.0068; M2: *r =* 0.91, *P =* 0.011). These results confirm that the perceptual improvements were highly orientation specific.

### Temporal dynamics of learning in V4

To determine how learning alters V4 representation of contours, we examined the temporal profile of population-averaged activity [poststimulus time histogram (PSTH)] across different training phases (Fig. 2, A to C and E to G, for the two animals, respectively). We divided the neural response into two distinct windows: an early, feedforward-dominated epoch (0 to 95 ms from stimulus onset) and a late, feedback-influenced epoch (95 to 500 ms).

**Fig. 2.**
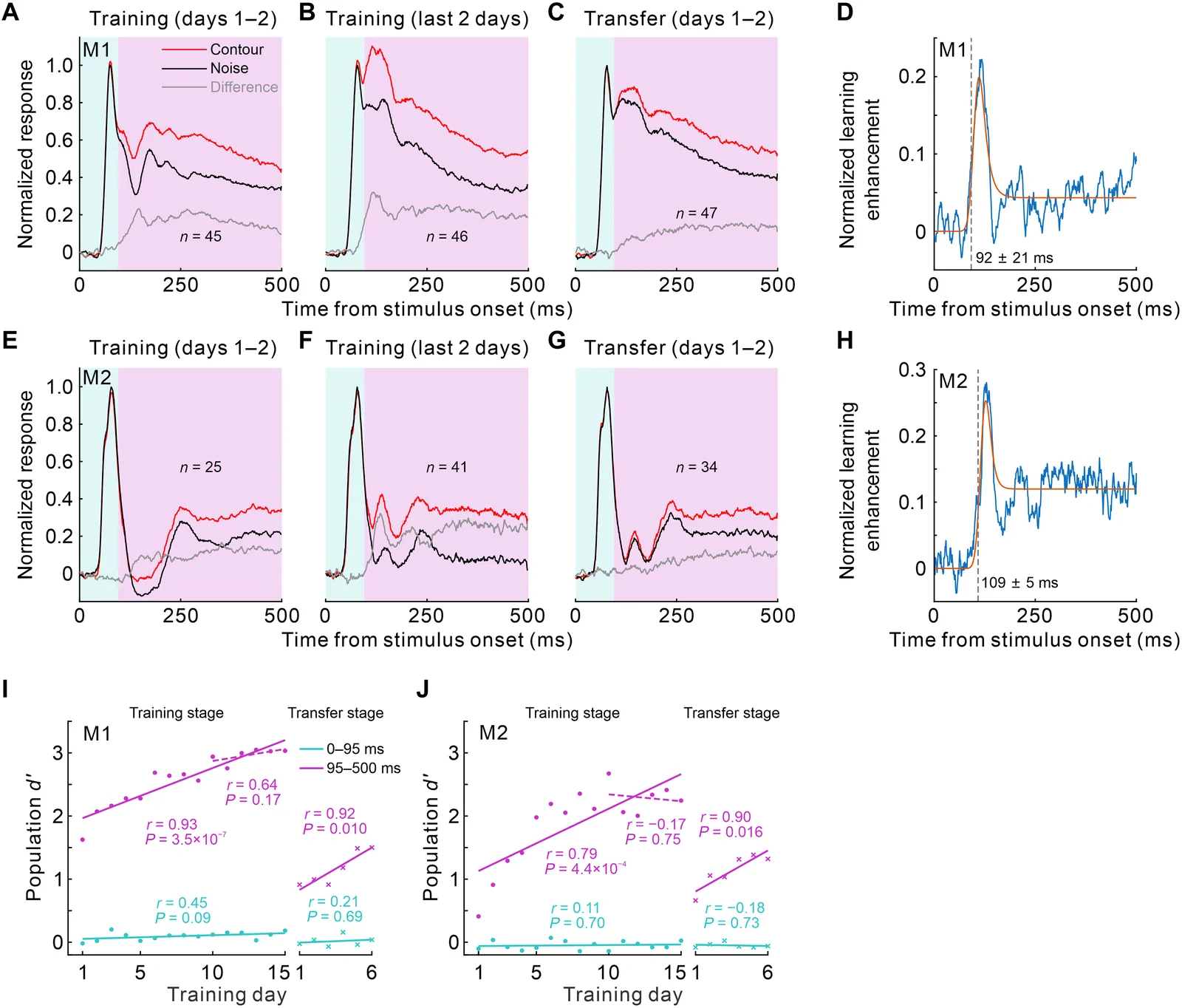
Learning effects in V4. (**A** to **C**) PSTHs averaged across valid sites (inset *n*; Materials and Methods) and over days in monkey M1. Responses to contour patterns (red; pooled across all 36 conditions) are normalized to the peak of corresponding responses to noise patterns (black). The contour signal (difference between contour and noise responses) is shown in gray. Time zero indicates stimulus onset. Cyan and magenta shading mark the early (0 to 95 ms) and late (95 to 500 ms) response epochs for subsequent data analysis. Data are shown for the first two training days (A), last two training days (B), and first two transfer days (C). Raw firing rate PSTHs are provided in fig. S1 (A to C). (**D**) Learning-induced enhancement (blue), computed by subtracting the contour signal in the first two training days [gray in (A)] from that in the last two training days [gray in (B)]. The vertical dashed line indicates the latency of learning-induced enhancement measured by an established curve fitting (orange) approach. Latency estimation using raw firing rates is shown in fig. S1D. (**E** to **H**) Similar to (A) to (D), for monkey M2 (see also fig. S1, E to H, based on raw firing rates). (**I**) Population decoding (*d′*) in monkey M1 during training (left) and transfer (right). Cyan and magenta symbols correspond to early and late response epochs, respectively. Solid lines show linear fits; the dashed cyan line represents a separate fit for the final 6 training days. Pearson correlation coefficients (*r*) and *P* values (*P*) are shown. Number of valid sites across training and transfer days: *n =* 43 to 47 (mean ± SD: 45.9 ± 1.3). (**J**) Similar to (I), for monkey M2. Valid sites: *n =* 18 to 39 (30.7 ± 5.2).

In the early epoch, neural responses to CP and NP were nearly indistinguishable in both monkeys, and this initial wave of activity remained remarkably stable throughout the training and transfer stages (Fig. 2, A to C and E to G). In contrast, the late response epoch exhibited a clear divergence between contour and noise responses. Crucially, the magnitude of this contour-induced enhancement grew substantially as training progressed. The difference between contour and noise responses (defined as the contour signal) was markedly larger in the final two training days compared to the first 2 days (Fig. 2, B versus A, F versus E), but this enhancement largely vanished when the contour was rotated by 90° (Fig. 2, C versus B, G versus F). We quantified this learning-induced enhancement as the difference in contour signal between the last two training days and the first 2 days (Fig. 2, D and H). For the two animals, respectively, the enhancement emerged at 92 ± 21 ms and 109 ± 5 ms (mean ± SD) after stimulus onset, closely matching the timing of the contour signal.

Previous work has shown that individual V4 neurons can encode global contours from response onset, but the contour signal is weaker during the early than the late response epoch; moreover, neural activity is highly selective for contour orientation and spatial position, with the strongest responses occurring when the contour is centered in the RF at the preferred orientation (*35*). Thus, when pooling neurons with heterogeneous RF-contour relationships, the population-averaged contour signals would be attenuated, especially during the early response epoch, as observed in the current study.

The temporal dissociation between early and late response epochs raises the question of how learning alters the information content of the V4 population across these two phases.

### Population sensitivity and orientation specificity

To quantify the information content of the V4 population, we used a Fisher linear discriminant (FLD; Materials and Methods) to decode contour presence from NPs. This allowed us to calculate a population *d′* as a measure of neural sensitivity (Fig. 2, I and J), taking into account not only the mean and variance of firing rates of individual V4 sites but also the structure of correlated responses.

The decoding analysis mirrored the temporal split observed in the firing rates. Within the early epoch (0 to 95 ms), population sensitivity remained flat and showed no training-related improvement (*d′* values indicated by horizontal cyan lines in Fig. 2, I and J). The population *d′* averaged across training and transfer days significantly differed from zero for both monkeys (M1: *P =* 0.0012; M2: *P =* 0.0033; two-tailed Wilcoxon signed-rank test, *n =* 21 days), suggesting that contour signals are already present in the early epoch, albeit weak and insensitive to training. By contrast, in the late epoch (95 to 500 ms), neural sensitivity increased steadily during the training stage (solid magenta lines, left panels), reached a plateau during the final 6 days (dashed magenta lines), and dropped sharply upon the 90° rotation of the stimulus. Consistent with the behavioral data, subsequent training at the new orientation led to a recovery of population *d′* (magenta lines, right panels). These results demonstrate that the neural signatures of learning in V4 are orientation-specific and restricted to the late-phase response.

In addition to the population *d′*, we also computed a sensitivity index (*d′*; fig. S2, A to D) for each V4 site to discriminate between CP and NP and averaged it across all sites. The result—at the level of individual sites by discounting correlated neuronal responses (fig. S3)—largely mirrors the results from population decoding (compare fig. S2, E and F, with Fig. 2, I and J), consistent with the view that grouping and segmentation in visual cortex are primarily encoded by neuronal firing rates (*36*).

The stable early neural responses and the late-epoch–specific learning effect were further validated via a representational similarity analysis (fig. S4), which showed that evolution in representational geometry was restricted to the late response epoch. These findings reveal a critical dissociation: Early feedforward responses remained stable across training, whereas late-phase activity accounted for the full extent of learning-related enhancement and behavioral specificity.

### Widespread enhancement across individual neurons

Because orientation-selective neurons in V4 exhibit clear orientation-dependent modulation in response to camouflage collinear contours (*35*), we next examined the relationship between learning-related enhancement and individual sites’ orientation selectivity, which was measured during an independent orientation-tuning block with grating stimuli.

We grouped V4 sites by orientation selectivity, quantified using the orthogonal-to-preferred response ratio (*O/P* ratio; Materials and Methods). Sites with *O/P* ≤ 0.7 were classified as orientation selective; *O/P* > 0.7 were categorized as low selectivity. The sampled V4 sites in the two monkeys exhibited markedly different distributions. In M1, the vast majority of sites were orientation selective (examples in Fig. 3, A and B; distribution in Fig. 3E). In M2, by contrast, most of the recorded sites fell into the low-selectivity category (examples in Fig. 3, C and D; distribution in Fig. 3G).

**Fig. 3.**
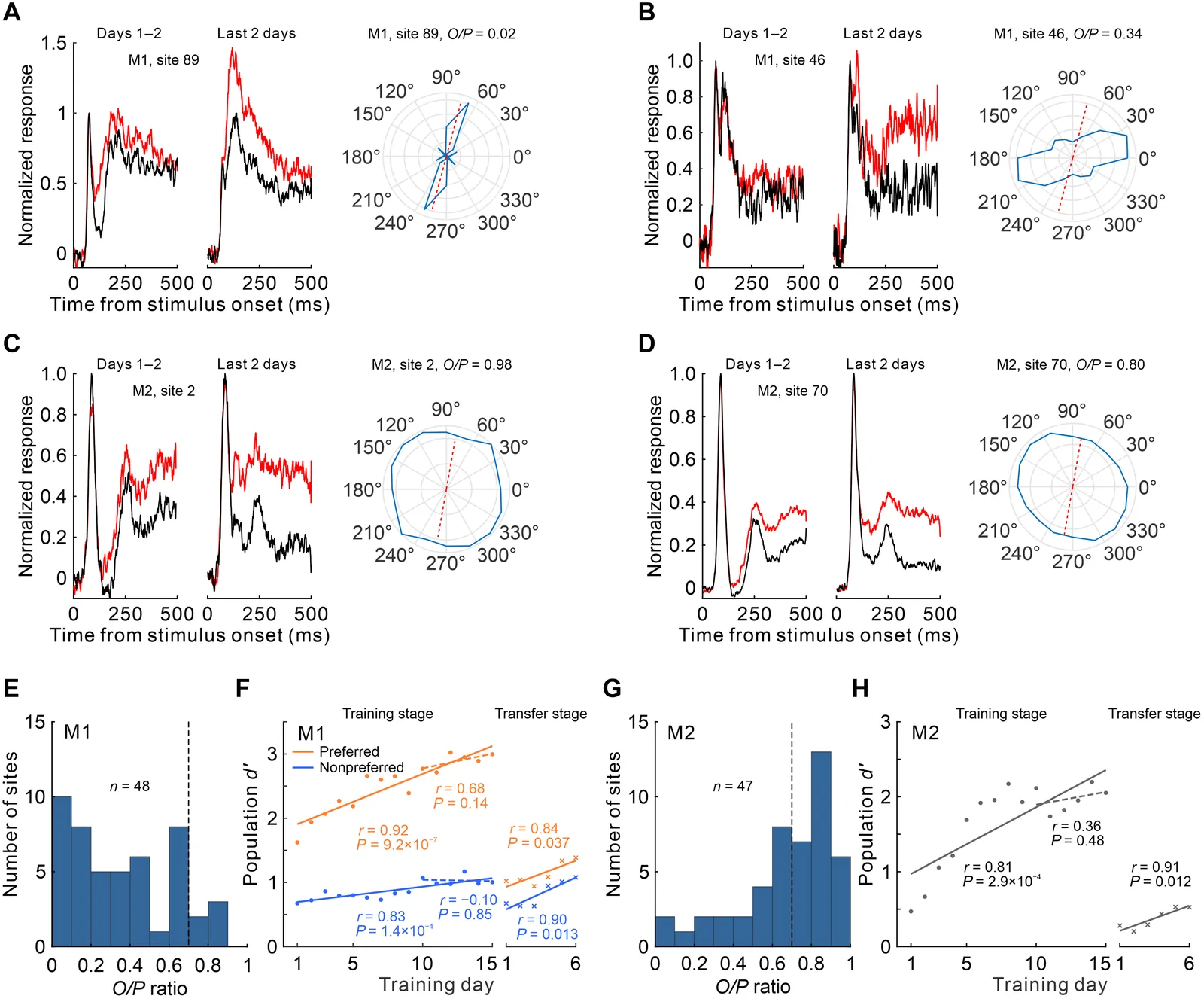
Dissociation between orientation-specific learning and widespread neural modulation in V4. (**A** to **D**) Example V4 sites exhibiting learning effects regardless of orientation selectivity. PSTHs show responses to contour patterns (red; averaged across 36 conditions) and noise patterns (black) for the first two (left) and last two (middle) training days. Normalized orientation tuning curves are shown as polar plots (right; dashed red line indicates the contour orientation). (A) A contour-preferred site from monkey M1. (B) A contour-nonpreferred site from M1. (C) An orientation-nonselective site from M2. (D) A weakly orientation-tuned site from M2. (**E**) Distribution of orientation selectivity in monkey M1, quantified by the *O/P* ratio (Materials and Methods). The vertical dashed line (*O/P* = 0.7) separates orientation-selective sites (*O/P* ≤ 0.7) from low-selectivity sites (*O/P* > 0.7). (**F**) Population decoding (*d′*) based on the late response epoch (95 to 500 ms) of orientation-selective sites in M1. These sites were categorized into two groups according to the angular difference between the preferred orientation and the contour: contour-preferred (<45°; orange) and contour-nonpreferred (≥45°; blue). Decoding performance for each group is plotted across training (left) and transfer (right) days. Note that the 90° contour rotation during transfer swapped group assignment for each site. Solid lines show linear fits across each stage; dashed lines show fits to the final 6 training days. Pearson correlation (*r*) and *P* value (*P*) are shown. Site counts across training and transfer: contour-preferred *n =* 21 to 23 (mean ± SD: 22.0 ± 0.4); contour-nonpreferred *n =* 18 to 20 (19.2 ± 0.8). (**G**) Same as (E) for monkey M2. (**H**) Similar to (F), showing population decoding based on low-selectivity sites (*O/P* > 0.7) in M2. Site counts across training and transfer: *n =* 10 to 21 (16.4 ± 3.2).

One critical question for understanding orientation specificity is whether plasticity is restricted to neurons whose preferred orientations match the trained stimulus. To address this, we categorized the orientation-selective sites in monkey M1 into two groups: contour-preferred (tuning within 45° of the contour; e.g., Fig. 3A) and contour-nonpreferred (tuning ≥45° from the contour; e.g., Fig. 3B).

Learning-related improvements in population *d′* were nearly identical across both groups. While contour-preferred sites maintained a higher baseline sensitivity [consistent with (*35*)], both groups exhibited robust and significant learning curves (Fig. 3F, left, solid lines; contour-preferred: *r =* 0.92, *P =* 9.2 × 10^−7^; contour-nonpreferred: *r =* 0.83, *P =* 1.4 × 10^−4^). Improvements plateaued during the final 6 training days (dashed lines; contour-preferred: *r =* 0.68, *P =* 0.14; contour-nonpreferred: *r =* −0.10, *P =* 0.85). Even after rotating the contour by 90°, which effectively swapped the group identities, both sets of neurons showed comparable relearning rates (Fig. 3F, right: new contour-preferred, *r =* 0.84, *P =* 0.037; new contour-nonpreferred, *r =* 0.90, *P =* 0.013). The magnitude of mean improvement rate averaged across the training and transfer stages did not differ significantly between preferred (two orange curves combined) and nonpreferred sites (two blue curves combined): *d′* increased by 60.2 ± 3.5% versus 54.8 ± 4.7%; mean ± SEM, *P =* 0.32, bootstrap. This indicates that V4 plasticity is distributed broadly across the orientation-selective population rather than being tuned to the task stimulus.

For monkey M2, after subdividing the orientation-selective sites (*n =* 8 to 18 per day, 14.3 ± 2.5 across days) into contour-preferred and contour-nonpreferred categories, the resulting sample sizes were too small for reliable decoding analysis. However, a notably larger proportion of low-selectivity sites were recorded from this animal. These neurons either exhibited extremely broad tuning or were virtually untuned for orientation (Fig. 3, C and D), which could be used for further testing of diffuse plasticity.

Despite their lack of inherent orientation preference, these low-selectivity sites displayed robust learning effects. Population decoding confirmed that these neurons, as a group, significantly improved their ability to discriminate the trained contour from noise (Fig. 3H; training stage: *r =* 0.81, *P =* 2.9 × 10^−4^; last 6 training days: *r =* 0.36, *P =* 0.48; transfer stage: *r =* 0.91, *P =* 0.012). Crucially, this improvement was also orientation-specific; the gains vanished upon stimulus rotation but recovered with subsequent training.

Together, these results demonstrate that the neural signatures of perceptual learning in V4 are unexpectedly diffuse. While behavioral improvements are strictly orientation specific, neural enhancements are evident across the entire population, a widespread modulation that aligns with the divergent anatomical architecture of corticocortical feedback (*30*–*33*). Crucially, the restriction of these enhancements to the late response phase, combined with their absence at untrained stimulus orientations, suggests that specific learning does not require a localized feedforward architecture. Instead, these temporal and population-level dynamics point toward a model where specificity emerges from the interaction between broad feedback and stable, tuned sensory inputs.

## DISCUSSION

Our results provide a mechanistic resolution to a central paradox in visual perceptual learning: how behaviorally specific improvements can emerge from neural enhancements that are diffuse at the population level. By demonstrating that learning-related changes are confined to late-phase V4 responses while early feedforward activity remains stable, our findings provide direct evidence to decouple behavioral specificity from feature-specific plasticity within early sensory circuits. This dissociation contends with traditional fine-tuning accounts and suggests a more complex hierarchical interaction than simple reweighting. To reconcile these observations, we propose a feedforward-gated feedback framework in which training potentiates higher-order representations that deliver diffuse feedback, yet this broad modulation is selectively gated by stable, feature-selective feedforward inputs linked to the trained stimulus. This hierarchical, bidirectional interaction allows the visual system to achieve refined expertise without compromising the stability of its core sensory filters.

### The feedforward-gated feedback framework

In the classical feedforward-specificity account (Fig. 4A), learning is thought to strengthen specific feature-tuned pathways. This model predicts that learning-related enhancement should be restricted to neurons whose preferred orientation matches the trained stimuli and should manifest during the initial wave of sensory processing. Our data contradict these predictions: Feedforward V4 responses were unaffected by training, and neural enhancement was observed even in neurons tuned to the orthogonal orientation.

**Fig. 4.**
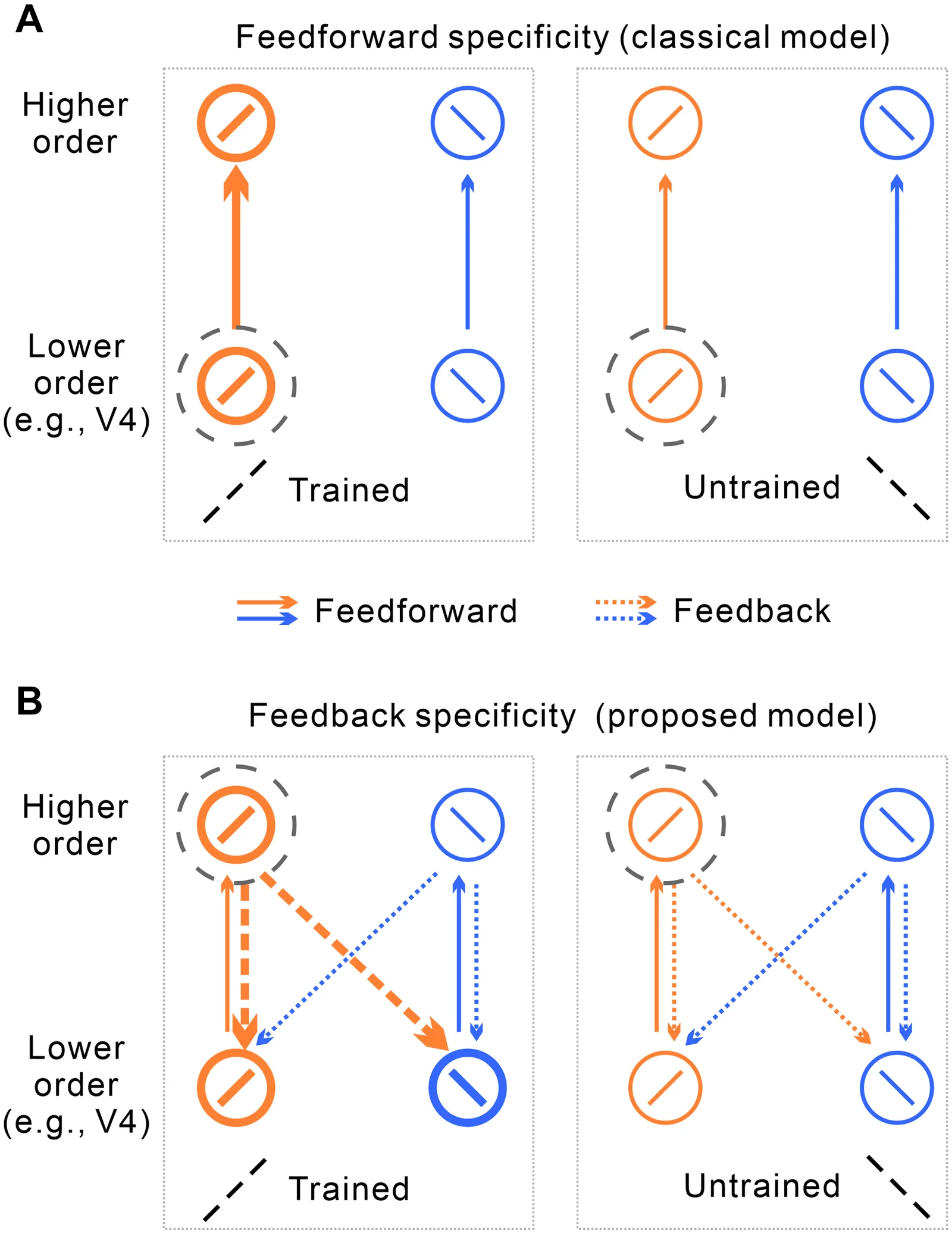
Circuit mechanisms underlying learning specificity. (**A**) Classical feedforward-specificity model. Neuronal nodes are represented by circles, with internal bars indicating orientation preference. Orange circles denote nodes tuned to the trained orientation; blue circles denote nodes tuned to the untrained orientation. Learning is assumed to induce local plasticity within feature-selective V4 neurons (indicated by gray dashed circle). Left: Presentation of the trained stimulus activates the strengthened V4 node and potentiates feedforward processing. Right: For untrained orientations, the strengthened V4 node remains inactive, preventing learning transfer. While this model predicts behavioral specificity, it contradicts our observation of widespread neural modulation across the V4 population. (**B**) Proposed feedforward-gated feedback framework. Learning primarily potentiates the representations in higher-order cortical nodes. Left: The trained stimulus activates the potentiated higher-order node, which delivers enhanced, diffuse feedback to the entire V4 population. This results in widespread neural enhancement observed in our data, spanning neurons with diverse tuning preferences. Right: Untrained orientations activate nonpotentiated higher-order nodes, precluding feedback-driven enhancement. In this framework, the stability of feature-selective feedforward inputs acts as a functional gate; diffuse feedback broadly recruits the early cortical population, but behavioral specificity is maintained because only the trained feedforward pathway is active to receive and “gate” this modulation. This mechanism allows the brain to achieve task-specific flexibility without compromising the long-term stability of core sensory representations.

Conversely, our feedforward-gated feedback framework (Fig. 4B) posits that learning primarily potentiates higher-order nodes. When the trained stimulus is presented, these potentiated nodes deliver enhanced, diffuse feedback to V4, boosting the entire population’s representation. Here, “diffuse” refers to a projection architecture that deviates from the high-specificity, feature-matched topography typical of feedforward pathways. Operationally, we define diffuse feedback as modulation that terminates across neurons with diverse stimulus preferences, rather than being restricted to iso-tuned populations, consistent with previous anatomical and neurophysiological studies (*30*–*33*). When an untrained orientation is presented, it activates a different, nonpotentiated higher-order representation, which provides no feedback boost. This framework naturally explains why learning-related enhancements are confined to the late response epoch and are specific to the trained stimulus, leading to behavioral specificity.

Higher-order areas contributing to the potentiated feedback may include not only higher visual regions that retain orientation selectivity [e.g., the posterior inferotemporal cortex, see (*29*)] but also association cortices involved in abstract representations. For instance, after subjects learn to categorize stimulus orientations, perceptual learning transfers to untrained orientations within the same category but not across the category boundaries (*37*). Such findings demonstrate that cognitive factors, such as categorical representations, can modulate the feature specificity of perceptual learning in a top-down manner. Within our framework, these higher-order category or decision nodes deliver diffuse feedback that broadly facilitates population representations in early visual areas, but does so selectively when the trained categorical representation is engaged—producing system-level specificity despite neuron-level generalization.

While the precise anatomical source of the learning-related feedback signals remains to be identified, feedback influences are expected to operate across multiple levels of the visual hierarchy. In V1, contour-related signals emerge selectively during late response epochs, show longer latencies than those observed in V4, and are largely independent of individual neurons’ orientation preference (*35*). Contour detection training has been shown to recruit an increasingly larger population of V1 neurons to represent contours, substantially amplifying overall signals (*19*). Consistent with a feedback origin, inactivation of V4 nearly abolishes these delayed V1 contour responses (*34*). Together, these findings support feedforward-gated feedback as a general principle underlying learning-related plasticity across the hierarchical visual cortex. Although the present study focused on area V4, we do not imply that V4 is uniquely responsible for feedback-dependent optimization of sensory representations. Other upstream and downstream areas may play similar roles, potentially depending on the specific task used during training.

### Task architecture and generalizability

The nature of visual perceptual learning may depend on the task architecture. Basic-feature tasks, such as grating orientation discrimination, may rely on fine-tuning of feature selective neurons (*13*), changes in correlated variability (*21*), or reductions in population response variability (*38*). These changes likely reflect local synaptic plasticity and can persist even under anesthesia (*18*). In contrast, context-dependent tasks, such as our camouflaged contour detection task, require the integration of information across the visual field, a process heavily dependent on top-down feedback modulation [for review, see (*39*)]. Causal interventions confirm that contextual interactions in early visual cortex critically depend on these feedback inputs (*34*, *40*–*42*). Learning in such tasks primarily engages the late components of neural responses and induces modulation of firing rates (*17*, *19*, *20*, *22*).

Therefore, our findings should not be interpreted as a general rejection of feedforward contributions to all forms of perceptual learning. Rather, they demonstrate that, for complex, context-dependent spatial integration, the brain uses a fundamentally different solution, namely, the feedforward-gated feedback framework proposed here. Whether learning-induced neural enhancement arises in the early or late response epoch likely represents a flexible axis that the brain adjusts according to task demands, favoring rapid local feature decoding or prolonged global contextual integration. This provides a more integrative and balanced framework for the perceptual learning field.

### Temporal interpretation and alternative explanations

Distinguishing feedforward and feedback influence solely by temporal epoch represents a limitation of the present study. We do not dispute that the late response epoch in V4 likely contains delayed feedforward or intrinsic recurrent components, nor do we claim that temporal latency alone definitively identifies feedback. However, our focus is specifically on the learning-induced modifications observed within this epoch. If these modifications were driven primarily by delayed feedforward inputs or local intracortical circuit optimization, the learning effects should be strictly constrained by each neuron’s baseline tuning geometry. Because contour encoding in V4 is highly orientation dependent (*35*), local or feedforward optimization would predict that enhancement is confined to units whose preferred orientations align with the stimulus contour. Our data directly contradict this prediction, supporting a feedback origin for the learning-related modulation.

Another potential alternative explanation is that the observed learning effects reflect enhanced spatial attention to the contour location. The latency of the learning-related enhancement observed here (as early as ∼90 ms; Fig. 2, D and H) substantially precedes the typical arrival of attentional modulation in V4 [∼170 ms, see (*43*, *44*)], providing a temporal signature that distinguishes learning-induced plasticity from purely endogenous attention. However, other studies have reported shorter attentional latencies (*45*, *46*), even if these early effects are modest in magnitude compared to late-stage modulation. The hypothesis that perceptual learning optimizes the strength, efficiency, or timing of top-down attentional routing and figure-ground segmentation mechanisms aligns well with our data. Under this view, the stable feedforward architecture ensures that sensory filtering properties remain uncompromised, while training systematically shapes downstream and feedback pathways to deploy task-relevant attentional and segmentation signals more rapidly and robustly. Nevertheless, attention may contribute as one component of a broader class of feedback signals. Within our framework, the principle of feedforward-gated feedback—namely, that diffuse top-down modulation is selectively engaged by particular input patterns—remains sufficient to explain how widespread neural modulation can coexist with stimulus-specific behavioral learning.

### Feedforward-gated feedback as a resolution to the stability-plasticity dilemma

Resolving the stability-plasticity dilemma—the need to maintain robust representations while adapting flexibly to environmental demands—presents a fundamental challenge for all distributed neural architectures (*47*). Modern systems neuroscience increasingly indicates that the brain handles this trade-off by preserving low-dimensional population dynamics even amidst localized structural or synaptic modifications (*48*). For instance, context-dependent visual and cognitive flexibility relies on the formation of specialized neural subspaces that route signals without disrupting underlying circuit stability (*49*). Our proposed feedforward-gated feedback framework extends these principles to early sensory processing hierarchies. We suggest that the brain leverages the topological stability of the feedforward stream to act as an anchor, selectively gating and contextualizing the plastic, broad adaptations delivered via diffuse top-down feedback.

By confining learning-related changes to the late, feedback-driven response phase, this mechanism recruits broad populations of neurons to represent task-relevant information while preserving stable sensory representations, effectively resolving the stability-plasticity dilemma. It allows the cortex to flexibly adapt to specific task demands without overwriting the fundamental filters required for general vision. In this view, feedback does not merely provide a nonspecific attentional boost; rather, it serves as a temporary scaffold that reconfigures early visual representations for the duration of a task, allowing neurons to multiplex their function in a state-dependent manner (*39*).

This mechanism also aligns with emerging frameworks of recurrent cortical processing (*50*–*52*), predictive coding (*53*, *54*), attentional modulation (*55*, *56*), and bottom-up–dependent gating (*57*). In these models, feedback signals are not merely additive or nonspecific; their expression is constrained by the feedforward sensory drive. Our framework extends these concepts by proposing that stimulus-driven bottom-up processes function as a computational gateway, where stable feedforward filters define the “context” (the trained stimulus) that allows the broad, diffuse feedback signal to become functional and specific.

### Interanimal variation as a test of robustness

The two monkeys showed markedly different distributions of orientation selectivity among V4 sites: Most sites in monkey M1 were clearly selective, whereas the opposite was true for monkey M2. This difference likely stems from the inhomogeneous, clustered organization of orientation selectivity in V4, where selective domains form functional columns spanning several hundred micrometers (*58*). As a result, a micrometer-scale shift in array placement can alter the ratio of orientation-selective to unselective or color-selective domains.

This sampling variation does not undermine but rather highlights the robustness and generalizability of our central findings: Despite different tuning distributions, both monkeys exhibited identical core phenomena—learning-induced enhancements emerged in the late response epoch and spread broadly across neurons, regardless of their orientation preference. Thus, the feedforward-gated feedback mechanism appears to be a consistent organizational principle across different subregions of the V4 cortical map.

### Implications for the readout versus plasticity debate

Our results provide a bridge between two traditionally opposing models: the sensory plasticity model, which posits localized changes in early cortical filters, and the reweighting model, which suggests optimized readout without altering early representations (*59*–*62*). The reweighting model effectively explains behavioral specificity through the selective weighting of stable sensory inputs. However, our observation of robust, learning-induced firing rate enhancements in V4 suggests that sensory representations are dynamic rather than fixed, and that readout and representation may be functionally intertwined: The optimization of higher-order areas manifests as a reciprocal modulation of visual cortex via feedback.

This feedback-driven representation model suggests that the high-level decision-maker does not just “listen” more closely to the sensory neurons; it actively reshapes their activity to highlight task-relevant information. Therefore, learning and its specificity are not isolated properties of one cortical area or a single process, but are emergent properties of hierarchical, bidirectional interactions.

## MATERIALS AND METHODS

Two adult male rhesus monkeys (*M. mulatta*, 6.5 to 9 kg; named M1 and M2) participated in this study. All procedures complied with the National Institutes of Health *Guide for the Care and Use of Laboratory Animals* and were approved by the Institutional Animal Care and Use Committee of Beijing Normal University [approval no. IACUC (BNU) - NKLCNL 2021-15].

### Surgical procedures and electrode implantation

Monkeys were initially anesthetized with ketamine (10 mg/kg, i.m.) and maintained under isoflurane anesthesia (1.5 to 2.5% in 100% O_2_) following intubation. A titanium headpost was affixed to the skull with bone screws to stabilize the head during training and recordings. After initial fixation training, a 6 × 8 microelectrode array (Blackrock Microsystems) was implanted into the superficial layers of area V4. Electrodes were 0.5 mm in length and spaced 0.4 mm apart.

### Contour stimuli and behavioral task

The stimulus and task design followed a previous study (*19*). Visual stimuli were generated with a ViSaGe system (Cambridge Research Systems) and displayed on a 22-inch (55.8-cm) CRT monitor (1200 × 900 pixels, 100 Hz refresh rate) at a viewing distance of 100 cm.

Stimuli were circular patterns (7.6° diameter for M1 and 8.5° for M2; Fig. 1, A to C) divided by a grid of hidden rhomboids (*63*). Each rhomboid compartment—which had equal base and height (0.4° for M1 and 0.5° for M2)—contained a small white bar (0.2° × 0.04° for M1 and 0.25° × 0.05° for M2). In the NP, the orientation of each bar was randomly assigned. In the CP, a collinear contour was formed by aligning three, five, or seven adjacent bars along a diagonal axis of the hidden grid, while the remaining bars were identical to those in the NP. The center-to-center spacing between collinear bars was adjusted by introducing a skew angle to the hidden rhomboid grid, defining six possible collinear spacings: 1.0, 1.2, 1.4, 1.6, 1.8, or 2.0 times the height of a rhomboid compartment. Contours could be embedded at one of two possible locations, positioned on opposite sides of the pattern center and separated by 1.0°. These settings resulted in 36 unique CPs (3 contour lengths × 6 collinear spacings × 2 possible locations), each with a corresponding NP.

Each trial began with a 0.1° fixation point at the screen center. Monkeys were required to maintain fixation within a 0.6° radius window. After 300 ms of stable fixation, a CP and its corresponding NP were simultaneously shown for 500 ms, followed by a 300-ms blank screen. Breaking fixation aborted the trial. After the blank period, two choice dots appeared at the prior stimulus locations; monkeys had 800 ms to saccade to the CP location to receive a juice reward. The CP and NP were randomly positioned to the left or right of the fixation point, with one pattern centered on the recorded RFs. To assess choice bias, 20% of trials were control trials featuring two identical NPs, with rewards delivered randomly. During the training stage (15 days), the contour orientation was fixed (75° for M1 and 80° for M2); in the transfer stage (6 days), the orientation was rotated by 90°.

### Behavioral pretraining and shaping

Before the formal onset of the camouflaged training sessions, both animals underwent a structured, multistep pretraining regimen. This shaping process included the following: (i) fixation training: animals were trained to maintain stable fixation within a strict 1.2° × 1.2° visual window; (ii) basic detection training: animals learned the behavioral rule of the task using high-contrast, uncamouflaged target contours presented on a blank background; these stimuli were displayed in the two visual-field quadrants orthogonal to those used for assessing learning effects; and (iii) introduction of noise: over multiple days, the luminance of the background noise elements was gradually increased while the luminance of the target contour was decreased. Once the animals could perform the task with the camouflaged (isoluminant) contours, the stimuli were moved to the quadrants intended for examining learning effects, and the formal training sessions began.

Day 1 of our formal dataset marks the very first session in which the animals were exposed to the specific, fixed target-contour configuration and background statistics used throughout the longitudinal learning study.

### Electrophysiological recording

Neural recordings were obtained using a Cerebus acquisition system (Blackrock Microsystems). The raw signal was band-pass filtered (250 to 7500 Hz), and spikes were detected at a voltage threshold of −3.5 × RMS of the background noise. All analyses were based on MUA.

Recording sites were included in the analysis only if they showed a significant visually evoked response to the NPs (*P <* 0.05, Wilcoxon signed-rank test comparing stimulus-evoked firing rates against spontaneous activity). The number of valid sites varied daily, ranging from 43 to 47 (mean ± SD: 45.9 ± 1.3) for M1 and 18 to 39 (30.7 ± 5.2) for M2. When pooling across all days, total nonrepetitive sites were 48 for M1 and 47 for M2. These daily fluctuations, with individual differences, are likely caused by (i) microscale variations in electrode impedance; (ii) subtle shifts in brain seating relative to the array grid; and (iii) daily adjustments of software spike-sorting thresholds to maintain consistent signal-to-noise criteria.

### V4 RF mapping

RFs were mapped using drifting square-wave gratings (2 cycles/°, drifting at 2 cycles/s) presented within narrow vertical or horizontal strips (0.3° × 7° or 7° × 0.3°) whose positions were varied horizontally or vertically. For each site, Gaussian functions were fitted to the response profiles along both axes. The RF extent was defined as an ellipse with major and minor axes corresponding to 2 × 1.96 SD of the respective Gaussian fits (Fig. 1, B and C).

### Orientation selectivity and preference

Tuning curves were measured using a circular patch of drifting square-wave gratings (6° diameter, 2 cycles/°, 2 cycles/s) across 16 drifting directions uniformly distributed over 360°. Responses to opposite drifting directions were combined to produce an orientation tuning curve, which was then averaged across days for each site. For visualization in polar coordinates (Fig. 3, A to D), the 180° tuning curve was duplicated to cover the full 360° angular space.

Orientation selectivity was quantified using an orthogonal-to-preferred (*O/P*) response ratio (*64*). We first identified the preferred orientation (*P*) as the maximum response from the raw tuning curve; the orthogonal orientation (*O*) was then defined relative to this preferred orientation. The *O/P* ratio was computed as the response at *O* divided by the response at *P*, with spontaneous baseline activity subtracted from both. Sites with *O/P* ≤ 0.7 were classified as orientation selective; those with *O/P* > 0.7 were categorized as low selectivity. For orientation-selective sites, we further fit the tuning curve with a Gaussian function for more accurate estimation of the preferred orientation, based on which the sites were categorized as contour-preferred or contour-nonpreferred. This *O/P* ratio threshold of 0.7 was empirically chosen to balance two critical requirements: (i) preventing contamination of the low-selectivity group by moderately tuned neurons, and (ii) preserving sufficient statistical power for subgroup analyses (contour-preferred versus contour-nonpreferred) given the limited sample sizes in longitudinal recordings.

### Poststimulus time histogram

For each site and stimulus pattern, a PSTH was constructed by binning spike trains in 1-ms intervals and averaging across trials. The PSTH was then smoothed with a 9-ms boxcar filter after subtracting mean spontaneous activity (−300 to 0 ms prestimulus period). We averaged the PSTHs across all CPs for comparison with noise-pattern responses.

For the example sites shown in Fig. 3 (A to D), raw PSTHs were averaged across days and then normalized such that the peak of the noise-pattern PSTH was unity. For the population averages in Fig. 2 (A to C and E to G), contour- and noise-pattern PSTHs were first averaged across days for each site, and then across the population to obtain raw population PSTHs (fig. S1). Normalized population PSTHs were derived by rescaling these raw population PSTHs such that the peak response to the NPs was unity.

### Time course of the learning-induced enhancement

On the basis of the PSTHs, we first computed the contour signal as the difference between neuronal responses to the CP and NP (gray curves in Fig. 2, A to C and E to G). We then quantified the learning-induced enhancement as the difference in contour signal between the last two training days and the first 2 days (Fig. 2, D and H). This differential signal, f(t), was fitted with the following function (*65*)

$$f\left(t\right)=d*\text{exp}\left(\mu \alpha +0.5\sigma^{2}\alpha^{2}-\alpha t\right)*G\left(t,\mu +\sigma^{2}\alpha ,\sigma \right)+c*G\left(t,\mu ,\sigma \right)$$

where G(t,μ,σ) is a cumulative Gaussian function. The onset latency of the learning-induced enhancement was defined as the time point at which the fitted curve reached 33% of its maximum amplitude. To estimate the SEM, we performed a bootstrap procedure by resampling V4 recording sites with replacement and repeating the fitting procedure 1000 times.

### Decoding V4 population responses

FLD analysis (*66*) was used to compute the population discriminability index (*d′*). For each condition and day, an FLD classifier was trained to discriminate between CP and NP trials. The FLD identified the projection axis that maximized the *d′* between the two response distributions. Population responses were preprocessed using principal components analysis, retaining the first 10 components. Performance was evaluated using 10-fold cross-validation by randomly dividing dataset into 10 subsets, using one set for testing and the others for training. After cycling through the subsets so that each subset was used once for testing, the mean performance was calculated.

The learning-related improvement rate was calculated as the percent change in population *d′* between the first and last days. Improvement rates from training and transfer stages were averaged to estimate overall changes for the entire neural population. Variance and *P* values were estimated using a bootstrapping procedure by repeating the above cross-validation procedures 100 times. Specifically, under the null hypothesis that the improvement rates were identical between contour-preferred and nonpreferred V4 sites, we obtained a bootstrapped distribution of the difference in improvement rate (contour-preferred − nonpreferred). The two-tailed *P* value was calculated as twice the minimum of the proportion of bootstrap iterations that yielded an improvement rate difference less than zero or greater than zero.

## References

[1] A. Fiorentini, N. Berardi, Perceptual learning specific for orientation and spatial frequency. Nature 287, 43–44 (1980). 10.1038/287043a0 7412873

[2] A. Karni, D. Sagi, Where practice makes perfect in texture discrimination: Evidence for primary visual cortex plasticity. Proc. Natl. Acad. Sci. U.S.A. 88, 4966–4970 (1991). 10.1073/pnas.88.11.4966 PMC51788 2052578

[3] A. Schoups, R. Vogels, G. A. Orban, Human perceptual learning in identifying the oblique orientation: Retinotopy, orientation specificity and monocularity. J. Physiol. 483, 797–810 (1995). 10.1113/jphysiol.1995.sp020623 PMC1157819 7776259

[4] M. Ahissar, S. Hochstein, Learning pop-out detection: Specificities to stimulus characteristics. Vision Res. 36, 3487–3500 (1996). 10.1016/0042-6989(96)00036-3 8977015

[5] B. A. Dosher, Z. L. Lu, Mechanisms of perceptual learning. Vision Res. 39, 3197–3221 (1999). 10.1016/s0042-6989(99)00059-0 10615491

[6] M. Ahissar, S. Hochstein, Task difficulty and the specificity of perceptual learning. Nature 387, 401–406 (1997). 10.1038/387401a0 9163425

[7] Z. Liu, D. Weinshall, Mechanisms of generalization in perceptual learning. Vision Res. 40, 97–109 (2000). 10.1016/s0042-6989(99)00140-6 10768045

[8] S. C. Hung, A. R. Seitz, Prolonged training at threshold promotes robust retinotopic specificity in perceptual learning. J. Neurosci. 34, 8423–8431 (2014). 10.1523/JNEUROSCI.0745-14.2014 PMC4061387 24948798

[9] T. Zhang, L. Q. Xiao, S. A. Klein, D. M. Levi, C. Yu, Decoupling location specificity from perceptual learning of orientation discrimination. Vision Res. 50, 368–374 (2010). 10.1016/j.visres.2009.08.024 19716377

[10] L.-Q. Xiao, J.-Y. Zhang, R. Wang, S. A. Klein, D. M. Levi, C. Yu, Complete transfer of perceptual learning across retinal locations enabled by double training. Curr. Biol. 18, 1922–1926 (2008). 10.1016/j.cub.2008.10.030 PMC3045109 19062277

[11] C.-T. Law, J. I. Gold, Reinforcement learning can account for associative and perceptual learning on a visual-decision task. Nat. Neurosci. 12, 655–663 (2009). 10.1038/nn.2304 PMC2674144 19377473

[12] B. A. Dosher, P. Jeter, J. J. Liu, Z.-L. Lu, An integrated reweighting theory of perceptual learning. Proc. Natl. Acad. Sci. U.S.A. 110, 13678–13683 (2013). 10.1073/pnas.1312552110 PMC3746919 23898204

[13] A. Schoups, R. Vogels, N. Qian, G. Orban, Practising orientation identification improves orientation coding in V1 neurons. Nature 412, 549–553 (2001). 10.1038/35087601 11484056

[14] R. E. Crist, W. Li, C. D. Gilbert, Learning to see: Experience and attention in primary visual cortex. Nat. Neurosci. 4, 519–525 (2001). 10.1038/87470 11319561

[15] G. M. Ghose, T. Yang, J. H. R. Maunsell, Physiological correlates of perceptual learning in monkey V1 and V2. J. Neurophysiol. 87, 1867–1888 (2002). 10.1152/jn.00690.2001 11929908

[16] W. Li, V. Piëch, C. D. Gilbert, Perceptual learning and top-down influences in primary visual cortex. Nat. Neurosci. 7, 651–657 (2004). 10.1038/nn1255 PMC1440483 15156149

[17] W. Li, V. Piech, C. D. Gilbert, Learning to link visual contours. Neuron 57, 442–451 (2008). 10.1016/j.neuron.2007.12.011 PMC2409109 18255036

[18] T. Hua, P. Bao, C.-B. Huang, Z. Wang, J. Xu, Y. Zhou, Z.-L. Lu, Perceptual learning improves contrast sensitivity of V1 neurons in cats. Curr. Biol. 20, 887–894 (2010). 10.1016/j.cub.2010.03.066 PMC2877770 20451388

[19] Y. Yan, M. J. Rasch, M. Chen, X. Xiang, M. Huang, S. Wu, W. Li, Perceptual training continuously refines neuronal population codes in primary visual cortex. Nat. Neurosci. 17, 1380–1387 (2014). 10.1038/nn.3805 25195103

[20] Y. Yan, L. Zhaoping, W. Li, Bottom-up saliency and top-down learning in the primary visual cortex of monkeys. Proc. Natl. Acad. Sci. U.S.A. 115, 10499–10504 (2018). 10.1073/pnas.1803854115 PMC6187116 30254154

[21] A. M. Ni, D. A. Ruff, J. J. Alberts, J. Symmonds, M. R. Cohen, Learning and attention reveal a general relationship between population activity and behavior. Science 359, 463–465 (2018). 10.1126/science.aao0284 PMC6571104 29371470

[22] T. S. Lee, C. F. Yang, R. D. Romero, D. Mumford, Neural activity in early visual cortex reflects behavioral experience and higher-order perceptual saliency. Nat. Neurosci. 5, 589–597 (2002). 10.1038/nn0602-860 12021764

[23] G. Huang, S. Ramachandran, T. S. Lee, C. R. Olson, Neural correlate of visual familiarity in macaque area V2. J. Neurosci. 38, 8967–8975 (2018). 10.1523/JNEUROSCI.0664-18.2018 PMC6191526 30181138

[24] T. Yang, J. H. R. Maunsell, The effect of perceptual learning on neuronal responses in monkey visual area V4. J. Neurosci. 24, 1617–1626 (2004). 10.1523/JNEUROSCI.4442-03.2004 PMC6730469 14973244

[25] S. Raiguel, R. Vogels, S. G. Mysore, G. A. Orban, Learning to see the difference specifically alters the most informative V4 neurons. J. Neurosci. 26, 6589–6602 (2006). 10.1523/JNEUROSCI.0457-06.2006 PMC6674023 16775147

[26] H. Z. Adab, R. Vogels, Practicing coarse orientation discrimination improves orientation signals in macaque cortical area V4. Curr. Biol. 21, 1661–1666 (2011). 10.1016/j.cub.2011.08.037 21962714

[27] M. Sanayei, X. Chen, D. Chicharro, C. Distler, S. Panzeri, A. Thiele, Perceptual learning of fine contrast discrimination changes neuronal tuning and population coding in macaque V4. Nat. Commun. 9, 4238 (2018). 10.1038/s41467-018-06698-w PMC6185947 30315163

[28] R. Jing, C. Yang, X. Huang, W. Li, Perceptual learning as a result of concerted changes in prefrontal and visual cortex. Curr. Biol. 31, 4521–4533.e3 (2021). 10.1016/j.cub.2021.08.007 34450086

[29] H. Z. Adab, I. D. Popivanov, W. Vanduffel, R. Vogels, Perceptual learning of simple stimuli modifies stimulus representations in posterior inferior temporal cortex. J. Cogn. Neurosci. 26, 2187–2200 (2014). 10.1162/jocn_a_00641 24702452

[30] K. S. Rockland, K. S. Saleem, K. Tanaka, Divergent feedback connections from areas V4 and TEO in the macaque. Vis. Neurosci. 11, 579–600 (1994). 10.1017/s0952523800002480 8038130

[31] S. Shipp, S. Zeki, The organization of connections between areas V5 and V2 in macaque monkey visual-cortex. Eur. J. Neurosci. 1, 333–354 (1989). 10.1111/j.1460-9568.1989.tb00799.x 12106143

[32] S. Shipp, S. Zeki, The organization of connections between areas V5 and V1 in macaque monkey visual-cortex. Eur. J. Neurosci. 1, 309–332 (1989). 10.1111/j.1460-9568.1989.tb00798.x 12106142

[33] D. D. Stettler, A. Das, J. Bennett, C. D. Gilbert, Lateral connectivity and contextual interactions in macaque primary visual cortex. Neuron 36, 739–750 (2002). 10.1016/s0896-6273(02)01029-2 12441061

[34] Y. Xin, Y. Yan, W. Li, A central and unified role of corticocortical feedback in parsing visual scenes. Nat. Commun. 16, 6930 (2025). 10.1038/s41467-025-62279-8 PMC12304261 40721589

[35] M. Chen, Y. Yan, X. Gong, C. D. Gilbert, H. Liang, W. Li, Incremental integration of global contours through interplay between visual cortical areas. Neuron 82, 682–694 (2014). 10.1016/j.neuron.2014.03.023 24811385

[36] P. R. Roelfsema, Solving the binding problem: Assemblies form when neurons enhance their firing rate-they don’t need to oscillate or synchronize. Neuron 111, 1003–1019 (2023). 10.1016/j.neuron.2023.03.016 37023707

[37] Q. Tan, Z. Wang, Y. Sasaki, T. Watanabe, Category-induced transfer of visual perceptual learning. Curr. Biol. 29, 1374–1378.e3 (2019). 10.1016/j.cub.2019.03.003 PMC6482054 30930042

[38] Y. A. Cheng, M. Sanayei, X. Chen, K. Jia, S. Li, F. Fang, T. Watanabe, A. Thiele, R. Y. Zhang, A neural geometry approach comprehensively explains apparently conflicting models of visual perceptual learning. Nat. Hum. Behav. 9, 1023–1040 (2025). 10.1038/s41562-025-02149-x PMC12106082 40164913

[39] C. D. Gilbert, W. Li, Top-down influences on visual processing. Nat. Rev. Neurosci. 14, 350–363 (2013). 10.1038/nrn3476 PMC3864796 23595013

[40] P. C. Klink, B. Dagnino, M. A. Gariel-Mathis, P. R. Roelfsema, Distinct feedforward and feedback effects of microstimulation in visual cortex reveal neural mechanisms of texture segregation. Neuron 95, 209–220.e3 (2017). 10.1016/j.neuron.2017.05.033 28625487

[41] L. Nurminen, S. Merlin, M. Bijanzadeh, F. Federer, A. Angelucci, Top-down feedback controls spatial summation and response amplitude in primate visual cortex. Nat. Commun. 9, 2281 (2018). 10.1038/s41467-018-04500-5 PMC5995810 29892057

[42] L. Kirchberger, S. Mukherjee, U. H. Schnabel, E. H. Van Beest, A. Barsegyan, C. N. Levelt, J. A. Heimel, J. A. M. Lorteije, C. Van der Togt, M. W. Self, P. R. Roelfsema, The essential role of recurrent processing for figure-ground perception in mice. Sci. Adv. 7, eabe1833 (2021). 10.1126/sciadv.abe1833 PMC8245045 34193411

[43] E. A. Buffalo, P. Fries, R. Landman, H. L. Liang, R. Desimone, A backward progression of attentional effects in the ventral stream. Proc. Natl. Acad. Sci. U.S.A. 107, 361–365 (2010). 10.1073/pnas.0907658106 PMC2806732 20007766

[44] J. Poort, F. Raudies, A. Wannig, V. A. F. Lamme, H. Neumann, P. R. Roelfsema, The role of attention in figure-ground segregation in areas V1 and V4 of the visual cortex. Neuron 75, 143–156 (2012). 10.1016/j.neuron.2012.04.032 22794268

[45] H. Zhou, R. Desimone, Feature-based attention in the frontal eye field and area V4 during visual search. Neuron 70, 1205–1217 (2011). 10.1016/j.neuron.2011.04.032 PMC3490686 21689605

[46] K. A. Sundberg, J. F. Mitchell, T. J. Gawne, J. H. Reynolds, Attention influences single unit and local field potential response latencies in visual cortical area V4. J. Neurosci. 32, 16040–16050 (2012). 10.1523/JNEUROSCI.0489-12.2012 PMC3711872 23136440

[47] G. R. Yang, X. J. Wang, Artificial neural networks for neuroscientists: A primer. Neuron 107, 1048–1070 (2020). 10.1016/j.neuron.2020.09.005 PMC11576090 32970997

[48] J. A. Gallego, M. G. Perich, R. H. Chowdhury, S. A. Solla, L. E. Miller, Long-term stability of cortical population dynamics underlying consistent behavior. Nat. Neurosci. 23, 260–270 (2020). 10.1038/s41593-019-0555-4 PMC7007364 31907438

[49] T. Flesch, K. Juechems, T. Dumbalska, A. Saxe, C. Summerfield, Orthogonal representations for robust context-dependent task performance in brains and neural networks. Neuron 110, 1258–1270.e11 (2022). 10.1016/j.neuron.2022.01.005 PMC8992799 35085492

[50] V. A. F. Lamme, P. R. Roelfsema, The distinct modes of vision offered by feedforward and recurrent processing. Trends Neurosci. 23, 571–579 (2000). 10.1016/s0166-2236(00)01657-x 11074267

[51] A. M. Bastos, W. M. Usrey, R. A. Adams, G. R. Mangun, P. Fries, K. J. Friston, Canonical microcircuits for predictive coding. Neuron 76, 695–711 (2012). 10.1016/j.neuron.2012.10.038 PMC3777738 23177956

[52] P. Fries, Rhythms for cognition: Communication through coherence. Neuron 88, 220–235 (2015). 10.1016/j.neuron.2015.09.034 PMC4605134 26447583

[53] R. P. Rao, D. H. Ballard, Predictive coding in the visual cortex: A functional interpretation of some extra-classical receptive-field effects. Nat. Neurosci. 2, 79–87 (1999). 10.1038/4580 10195184

[54] K. Friston, A theory of cortical responses. Philos. Trans. R. Soc. Lond. B Biol. Sci. 360, 815–836 (2005). 10.1098/rstb.2005.1622 PMC1569488 15937014

[55] G. Spyropoulos, M. Schneider, J. van Kempen, M. A. Gieselmann, A. Thiele, M. Vinck, Distinct feedforward and feedback pathways for cell-type specific attention effects. Neuron 112, 2423–2434.e7 (2024). 10.1016/j.neuron.2024.04.020 PMC7616856 38759641

[56] J. D. Semedo, A. I. Jasper, A. Zandvakili, A. Krishna, A. Aschner, C. K. Machens, A. Kohn, B. M. Yu, Feedforward and feedback interactions between visual cortical areas use different population activity patterns. Nat. Commun. 13, 1099 (2022). 10.1038/s41467-022-28552-w PMC8888615 35232956

[57] L. B. Ekstrom, P. R. Roelfsema, J. T. Arsenault, G. Bonmassar, W. Vanduffel, Bottom-up dependent gating of frontal signals in early visual cortex. Science 321, 414–417 (2008). 10.1126/science.1153276 PMC3011100 18635806

[58] H. Tanigawa, H. D. Lu, A. W. Roe, Functional organization for color and orientation in macaque V4. Nat. Neurosci. 13, 1542–1548 (2010). 10.1038/nn.2676 PMC3005205 21076422

[59] B. A. Dosher, Z.-L. Lu, Perceptual learning reflects external noise filtering and internal noise reduction through channel reweighting. Proc. Natl. Acad. Sci. U.S.A. 95, 13988–13993 (1998). 10.1073/pnas.95.23.13988 PMC25004 9811913

[60] C.-T. Law, J. I. Gold, Neural correlates of perceptual learning in a sensory-motor, but not a sensory, cortical area. Nat. Neurosci. 11, 505–513 (2008). 10.1038/nn2070 PMC2424192 18327253

[61] B. A. Dosher, Z.-L. Lu, Hebbian reweighting on stable representations in perceptual learning. Learn. Percept. 1, 37–58 (2009). 10.1556/LP.1.2009.1.4 PMC2842576 20305755

[62] B. Dosher, Z.-L. Lu, Visual perceptual learning and models. Annu. Rev. Vis. Sci. 3, 343–363 (2017). 10.1146/annurev-vision-102016-061249 PMC6691499 28723311

[63] W. Li, C. D. Gilbert, Global contour saliency and local colinear interactions. J. Neurophysiol. 88, 2846–2856 (2002). 10.1152/jn.00289.2002 12424317

[64] K. R. Gegenfurtner, D. C. Kiper, S. B. Fenstemaker, Processing of color, form, and motion in macaque area V2. Vis. Neurosci. 13, 161–172 (1996). 10.1017/s0952523800007203 8730997

[65] P. S. Khayat, A. Pooresmaeili, P. R. Roelfsema, Time course of attentional modulation in the frontal eye field during curve tracing. J. Neurophysiol. 101, 1813–1822 (2009). 10.1152/jn.91050.2008 19176609

[66] R. A. Fisher, The use of multiple measurements in taxonomic problems. Ann. Eugen. 7, 179–188 (1936).

