# Supplementary material for: Specific expertise from diffuse feedback: A feedforward-gated mechanism
Source: Sci Adv. 2026 Oct 7;12(41):eaeh5972. doi: 10.1126/sciadv.aeh5972 (PMC13644753; doi:10.1126/sciadv.aeh5972)
Supplement: Supplementary file 1 [file sciadv.aeh5972_sm.pdf]

Science Advances
AAAS

Supplementary Materials for

Specific expertise from diffuse feedback: A feedforward-gated mechanism

Ruining Guo *et al.*

Corresponding author: Wu Li, liwu@bnu.edu.cn; Yin Yan, yanyin@bnu.edu.cn

Sci. Adv. **12**, eaeh5972 (2026)
DOI: 10.1126/sciadv.aeh5972

This PDF file includes:

Figs. S1 to S5

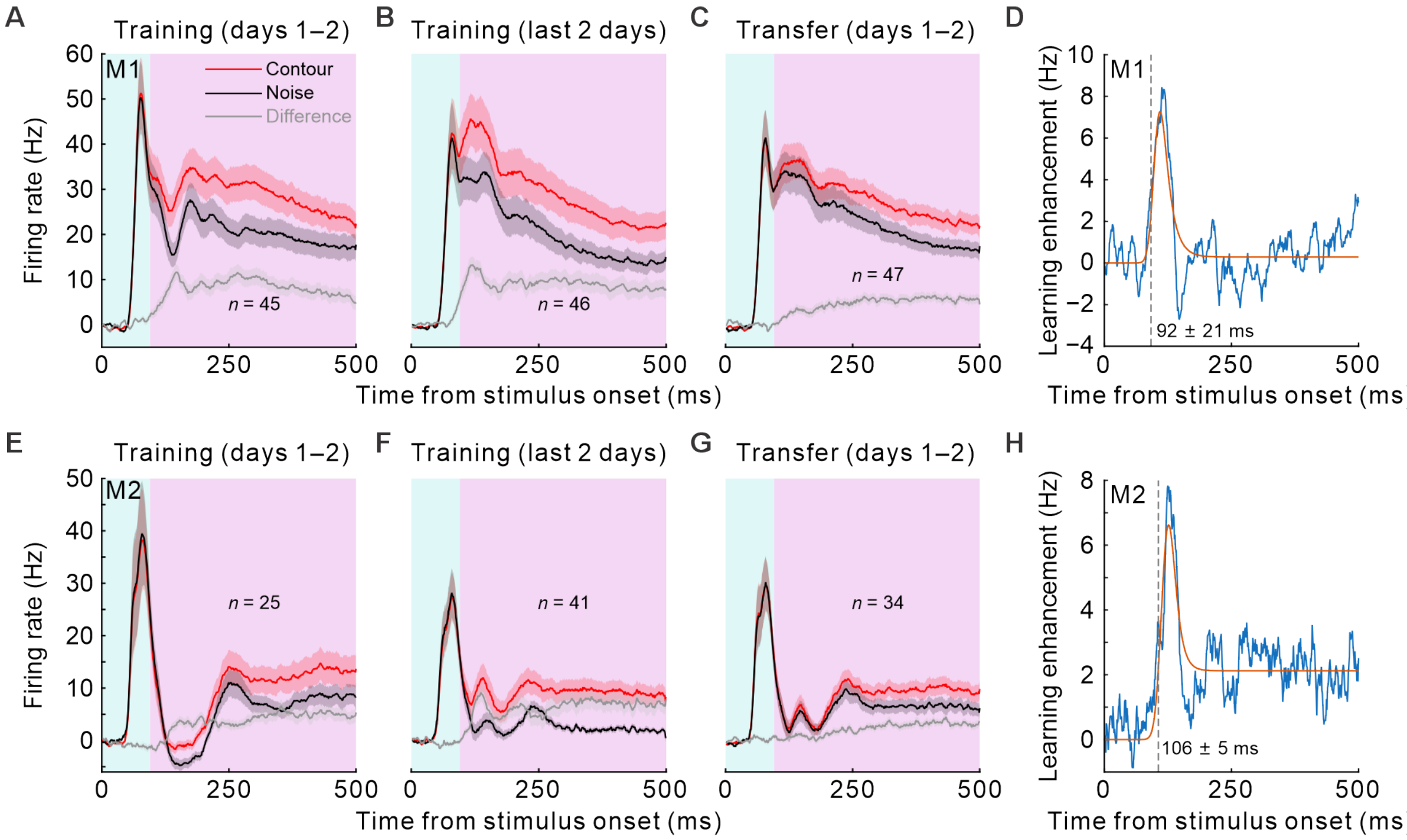

Fig. S1. Raw firing-rate post-stimulus time histograms (PSTHs) corresponding to the normalized data in Fig. 2, A to H.

(**A** to **C**) Population-averaged responses to contour patterns (red) and noise patterns (black) in Monkey M1, along with their differences (gray), are shown for the first two training days, last two training days, and first two transfer days. Cyan and magenta background shading mark the early (0–95 ms) and late (95–500 ms) response epochs. Shaded regions indicate SEM across sites (*n*).

(**D**) Learning-induced enhancement (blue), computed by subtracting the contour signal in the first two training days (gray in A) from that in the last two training days (gray in B). Vertical dashed line indicates the latency of learning-induced enhancement measured by an established curve fitting (orange) approach (see Materials and Methods).

(**E** to **H**) Similar to (A to D), for monkey M2.

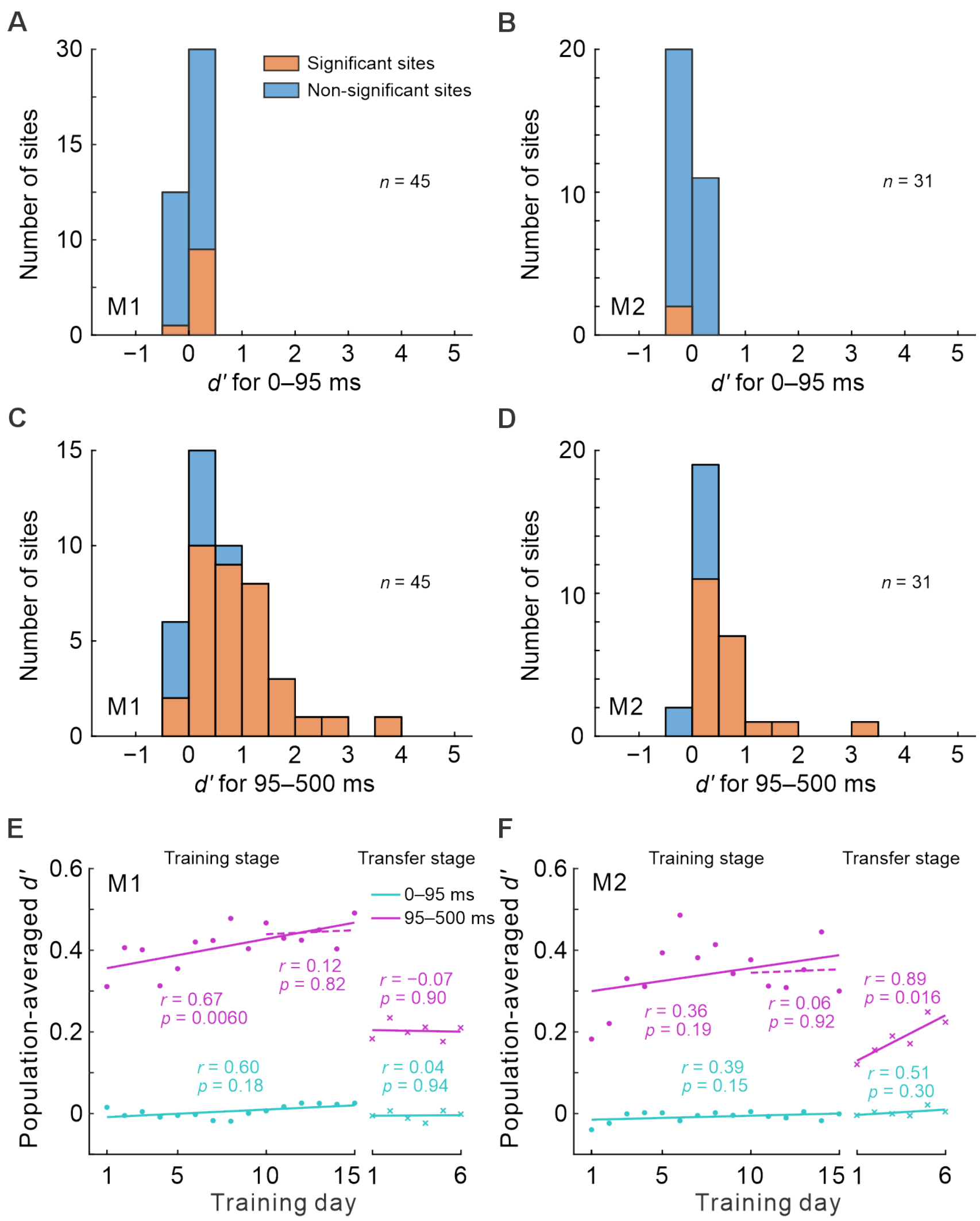

Fig. S2. Distribution of contour detection sensitivity (*d′*) across V4 sites and its training-related changes.

(**A**, **B**) Distribution of d' across V4 sites in early response epoch (0–95 ms) for monkey M1 (A) and monkey M2 (B). Each site's d' was calculated as: $d' = (\mu_{contour} - \mu_{noise})/\sqrt{(\sigma^2_{contour} + \sigma^2_{noise})}$, where μ and σ denote the mean and standard deviation of firing rates in response to contour and noise patterns, respectively, as indicated by the subscripts. Only trials from salient contour conditions (behavioral performance >95%; n = 13 conditions for M1, n = 16 for M2) on the last training day were included. Orange bars indicate sites with condition-averaged d' significantly different from zero (two-tailed *t*-test, $p < 0.05$); blue bars indicate sites not significantly different.

(**C**, **D**) Same as (A, B) but for the late response epoch (95–500 ms).

(**E**, **F**) Mean d' averaged across V4 sites for monkey M1 (E) and monkey M2 (F) during the training (left) and transfer (right) stages. Color and symbol conventions follow Fig. 2, I and J.

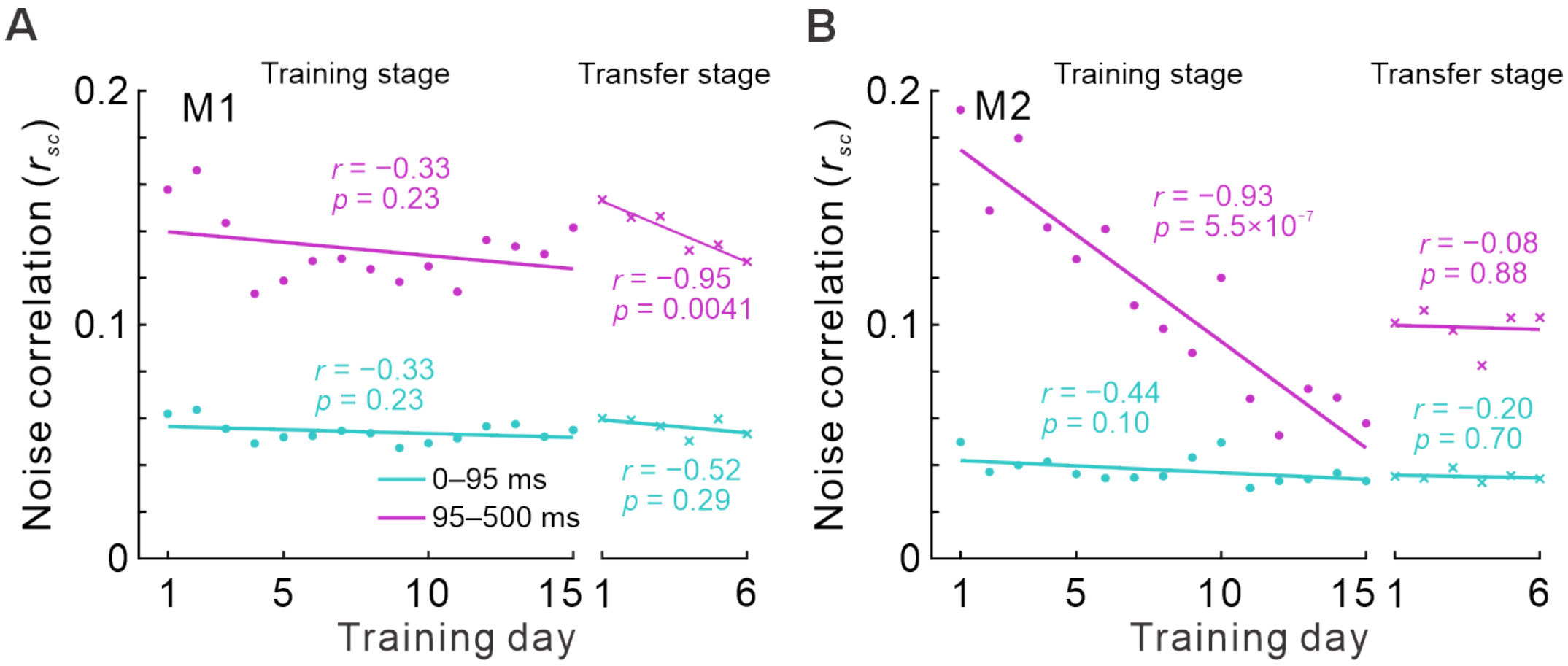

Fig. S3. Training-related changes in noise correlation.

(**A**) Population-averaged noise correlation across training and transfer stages for monkey M1. For each stimulus pattern, noise correlation was computed as the Pearson correlation coefficient between trial-by-trial spike counts of two V4 sites, and was then averaged across all site pairs and conditions for each day. Cyan and magenta symbols represent the early (0–95 ms) and late (95–500 ms) response epochs, respectively.

(**B**) Same as (A) but for monkey M2.

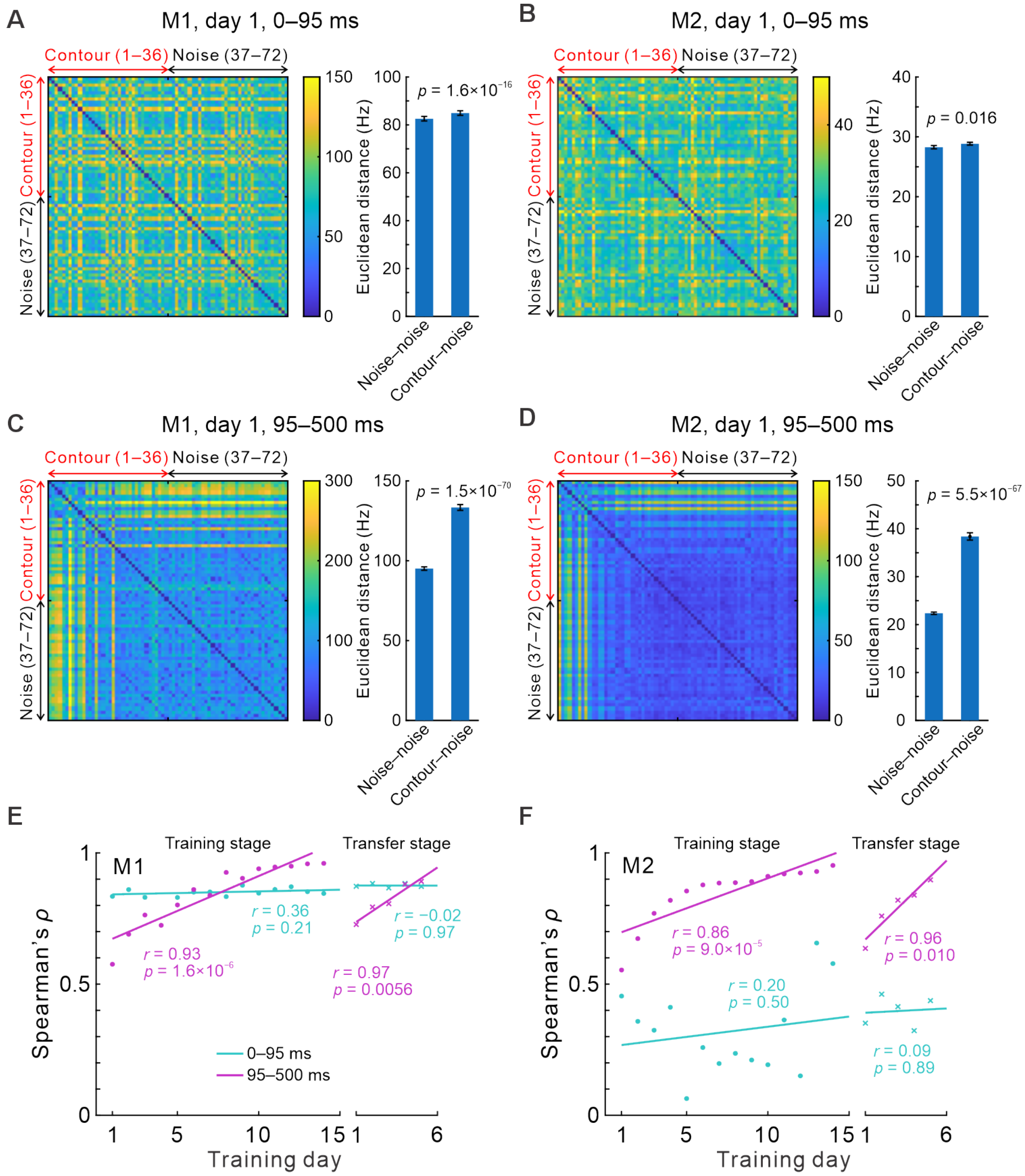

Fig. S4. Representational similarity analysis (RSA) and its training-related changes.

(**A**, **B**) Representational dissimilarity matrix (RDM) for the early response epoch (0–95 ms) on the first training day for monkey M1 (A) and monkey M2 (B). The RDM was constructed by computing the Euclidean distance between population response vectors for each stimulus pattern (36 distinct contour patterns and their corresponding noise patterns; see Materials and Methods) in neural population space, where each dimension represents the firing rate of a V4 site. RDM values are shown in pseudocolor (left). To test whether contour signals were present, we compared two sets of pairwise distances from the RDM: (1) distances among noise patterns (upper triangle of the lower-right quadrant, excluding the diagonal), and (2) distances between contour and noise patterns (upper-right quadrant, symmetrized by averaging with its transpose,

then taking the upper triangle, also excluding the diagonal). Significantly larger distances between contour and noise than within noise would indicate that the two stimulus classes evoke distinguishable population responses, as shown in the bar plot (right) for both monkeys (inset *p*-values, two-tailed Wilcoxon signed-rank test). This indicates that contour signals were already present on the first training day.

(**C**, **D**) Same as (A, B) but for the late response epoch (95–500 ms).

(**E**, **F**) Representational similarity across training and transfer days, referenced to the final day of each stage, for monkey M1 (E) and monkey M2 (F). The RDM from each day was compared to the reference RDM by computing Spearman's ρ (rank correlation) between their upper-triangular elements (excluding the diagonal). Cyan and magenta symbols represent similarity based on the early (0–95 ms) and late (95–500 ms) epochs, respectively. During the training and transfer stages, late-epoch representational similarity relative to the reference RDM increased progressively, indicating that activity patterns became aligned with those on the final training day. No such trend was observed in the early epoch. Instead, early-epoch similarity remained stable across days for monkey M1 but fluctuated substantially at a much lower level for monkey M2. This inter-animal difference reflects the underlying RDM structures: those from the early epoch remained nearly invariant for M1 but showed greater fluctuation for M2. This likely reflects that, although early-epoch contour signals were barely discernible in both monkeys, they were even weaker in M2 (Fig. 2, compare E to G with A to C), consistent with the general lack of orientation selectivity among V4 sites in this animal (Fig. 3, G vs. E). Thus, in M1, weak but consistent contour signals across daily RDMs yielded high and stable correlations, whereas in M2, even weaker and less stable signals produced lower and more variable correlations.

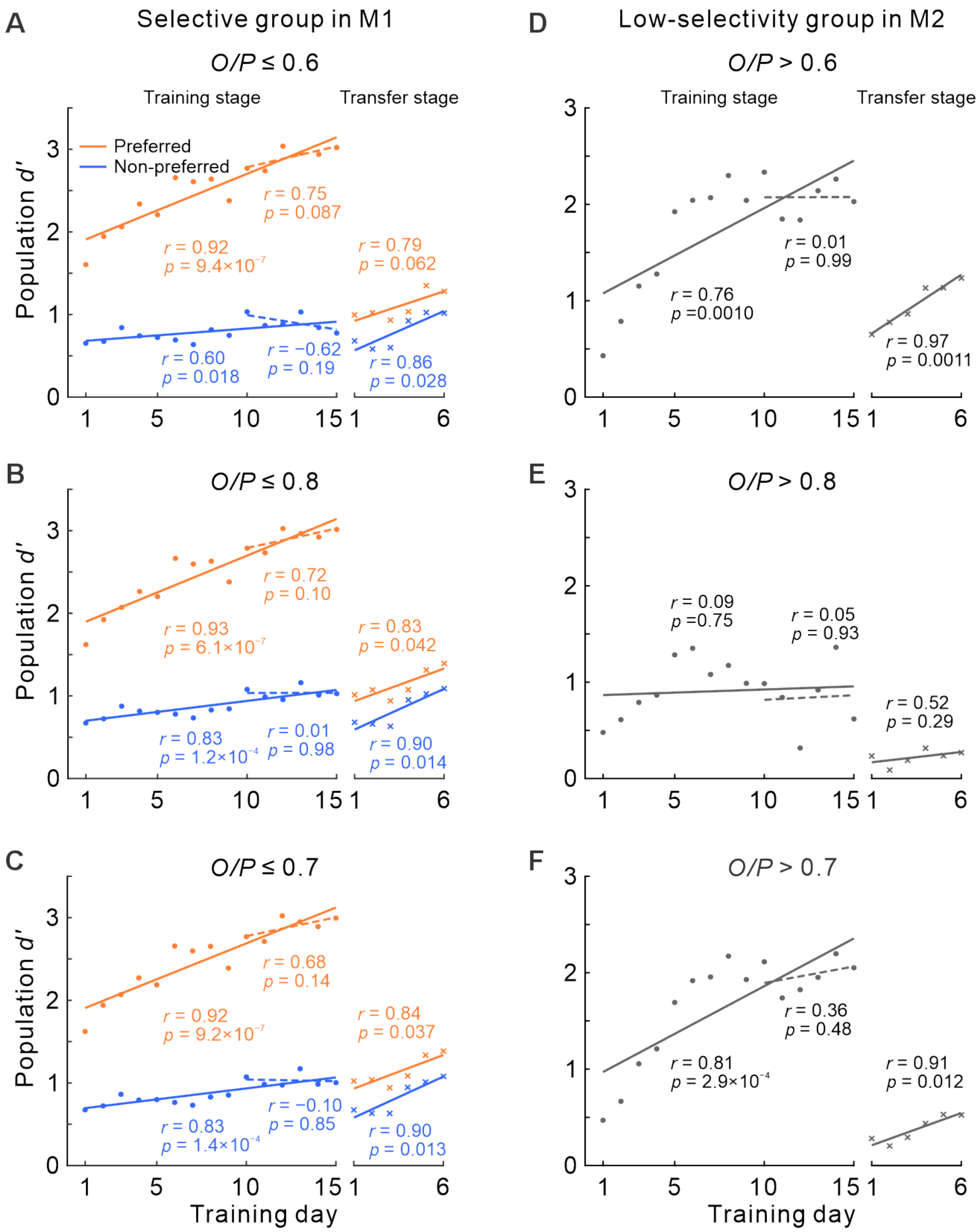

Fig. S5. Robustness of learning effects under alternative orientation selectivity thresholds, related to Fig. 3, F and H.

Left column: Population decoding (d') in monkey M1 based on the late response epoch (95–500 ms) of orientation-selective V4 sites, classified using three O/P thresholds (A: ≤ 0.6; B: ≤ 0.8; C: ≤ 0.7, the same as Fig. 3F). The sites meeting each criterion were further categorized into two groups according to the angular difference between the preferred orientation and the contour: contour-preferred (< 45°; orange) and contour-nonpreferred (≥ 45°; blue). Decoding performance for each group is plotted across days for the training and transfer stages. Note that the 90° contour rotation in the transfer stage swapped the group assignment for each site. Solid lines

show linear fits across each stage; dashed lines show fits to the final six training days. Pearson correlation coefficients (*r*) and *p*-values are shown.

Right column: population decoding in monkey M2 based on V4 sites with low orientation selectivity, classified using three *O/P* thresholds (D: *O/P* > 0.6; E: *O/P* > 0.8; F: *O/P* > 0.7, the same as Fig. 3H).

Sample sizes: The number of V4 sites (range and mean ± SD) contributing to each longitudinal analysis (across training and transfer) is shown below, where N_{prefer}, $N_{nonprefer}$, and N_{low} denote the numbers of contour-preferred, contour-nonpreferred, and low-selectivity sites, respectively.

(**A**) *O/P* ≤ 0.6: N_{prefer} = 19 to 21 (20.0 ± 0.4), $N_{nonprefer}$ = 12 to 14 (13.2 ± 0.8).

(**B**) *O/P* ≤ 0.8: N_{prefer} = 22 to 24 (23.0 ± 0.4), $N_{nonprefer}$ = 19 to 21 (20.2 ± 0.8).

(**C**) *O/P* ≤ 0.7: N_{prefer} = 21 to 23 (22.0 ± 0.4), $N_{nonprefer}$ = 18 to 20 (19.2 ± 0.8).

(**D**) *O/P* > 0.6: N_{low} = 12 to 29 (21.6 ± 4.6).

(**E**) *O/P* > 0.8: N_{low} = 8 to 15 (11.5 ± 2.0).

(**F**) *O/P* > 0.7: N_{low} = 10 to 21 (16.4 ± 3.2).

Summary: Across all *O/P* thresholds, the late-epoch, population-wide neural enhancement remained qualitatively similar, consistently showing positive correlation with training days, a drop on the initial transfer day, and subsequent relearning. The *O/P* threshold of 0.7 used in the main text (Fig. 3, F and H; duplicated here as C and F) was empirically chosen to balance two critical requirements: preventing contamination and preserving statistical power. We sought to minimize the probability of misclassifying neurons with moderate orientation tuning into the low-selectivity group (higher *O/P*), ensuring that our insights regarding the low-selectivity population remain clean and unconfounded. Additionally, because orientation-selective sites (lower *O/P*) must be further subdivided into contour-preferred and contour-nonpreferred groups to track longitudinal learning dynamics, it was critical to retain a sufficient and comparable number of sites within each group to maintain statistical robustness and obtain reliable decoding results. With the chosen threshold of *O/P* = 0.7, the sample size was well balanced between groups (C and F). By contrast, lower (A and D) or higher (B and E) thresholds caused a substantial (~30%) decrease in sample size for some groups ($N_{nonprefer}$ in A vs. C; N_{low} in E vs. F), compromising decoding reliability and statistical power.
